# Multiparametric hippocampal signatures for early diagnosis of Alzheimer's disease using ^18^F‐FDG PET/MRI Radiomics

**DOI:** 10.1111/cns.14539

**Published:** 2023-11-30

**Authors:** Zhigeng Chen, Sheng Bi, Yi Shan, Bixiao Cui, Hongwei Yang, Zhigang Qi, Zhilian Zhao, Ying Han, Shaozhen Yan, Jie Lu

**Affiliations:** ^1^ Department of Radiology and Nuclear Medicine, Xuanwu Hospital Capital Medical University Beijing China; ^2^ Beijing Key Laboratory of Magnetic Resonance Imaging and Brain Informatics Beijing China; ^3^ Key Laboratory of Neurodegenerative Diseases Ministry of Education Beijing China; ^4^ Department of Neurology, Xuanwu Hospital Capital Medical University Beijing China

**Keywords:** Alzheimer's disease, early diagnosis, hippocampal radiomics, machine learning, PET/MRI

## Abstract

**Purpose:**

This study aimed to explore the utility of hippocampal radiomics using multiparametric simultaneous positron emission tomography (PET)/magnetic resonance imaging (MRI) for early diagnosis of Alzheimer's disease (AD).

**Methods:**

A total of 53 healthy control (HC) participants, 55 patients with amnestic mild cognitive impairment (aMCI), and 51 patients with AD were included in this study. All participants accepted simultaneous PET/MRI scans, including ^18^F‐fluorodeoxyglucose (^18^F‐FDG) PET, 3D arterial spin labeling (ASL), and high‐resolution T1‐weighted imaging (3D T1WI). Radiomics features were extracted from the hippocampus region on those three modal images. Logistic regression models were trained to classify AD and HC, AD and aMCI, aMCI and HC respectively. The diagnostic performance and radiomics score (Rad‐Score) of logistic regression models were evaluated from 5‐fold cross‐validation.

**Results:**

The hippocampal radiomics features demonstrated favorable diagnostic performance, with the multimodal classifier outperforming the single‐modal classifier in the binary classification of HC, aMCI, and AD. Using the multimodal classifier, we achieved an area under the receiver operating characteristic curve (AUC) of 0.98 and accuracy of 96.7% for classifying AD from HC, and an AUC of 0.86 and accuracy of 80.6% for classifying aMCI from HC. The value of Rad‐Score differed significantly between the AD and HC (*p* < 0.001), aMCI and HC (*p* < 0.001) groups. Decision curve analysis showed superior clinical benefits of multimodal classifiers compared to neuropsychological tests.

**Conclusion:**

Multiparametric hippocampal radiomics using PET/MRI aids in the identification of early AD, and may provide a potential biomarker for clinical applications.

## Graphical abstract

This study aimed to investigate multiparametric imaging of the hippocampal radiomics for early diagnosis of AD using simultaneous PET/MRI. Our findings demonstrated that multi‐dimensional imaging of the hippocampal radiomics benefits the identification of early AD (aMCI especially) and may provide a potential biomarker for clinical applications in AD.

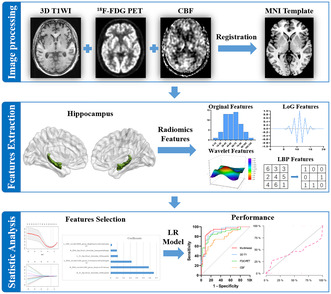

## 1 INTRODUCTION

Alzheimer's disease (AD) typically presents with gradually worsening amnesia, which is characterized by an early distribution of predominantly neurofibrillary tangles pathology within the medial temporal lobe. Mild cognitive impairment (MCI) is often viewed as the early stage of AD,^1^, ^2^ with amnestic MCI (aMCI) exhibiting a higher risk of progression to AD.^3^, ^4^ Therefore, it is crucial to detect preclinical AD early on to delay cognitive decline and slow down the progression of AD pathology.^5^, ^6^, ^7^, ^8^ Currently, available biomarkers allow for effective identification of patients with AD in the early stages, making individualized clinical intervention possible. However, the existing biomarkers are not sufficiently convenient and generalizable.

The hippocampus, a structure responsible for memory storage, is one of the most susceptible hallmarks of AD. Previous studies have achieved a high sensitivity of 84.0% and specificity of 84.0% in distinguishing AD from healthy control (HC) based on morphological features of the hippocampus.^9^, ^10^, ^11^ In addition to volume measurement, alterations in hippocampal glucose metabolism and blood flow have also been reported in AD and MCI patients, providing an additional information for diagnosis.^12^, ^13^ ^18^F‐fluorodeoxyglucose positron emission tomography (^18^F‐FDG PET) is a superior examination to evaluate hypometabolism induced by cognitive decline.^14^, ^15^ A recent study revealed that glucose metabolism decreased in the hippocampus bilaterally in AD patients.^16^ Furthermore, cerebral blood flow (CBF) of arterial spin labeling (ASL)‐magnetic resonance imaging (MRI) is believed to be tightly coupled to glucose metabolism and is a reliable indicator of hippocampal atrophy.^17^ However, single‐level features such as volume are insufficient for accurately characterizing the hippocampus as a generalizable biomarker. This limitation has led many studies to focus on characterizing more features through radiomics analysis.

Radiomics is an emerging image analysis method that captures the heterogeneity of tissue to provide valuable information for disease diagnosis. Subtle changes that cannot be detected visually in neuroimaging can be analyzed by extracting high‐throughput quantitative features through radiomics. Recent studies in structural MRI have demonstrated that hippocampal radiomics features have the potential as biomarkers for AD classification.^18^ In a multicenter study, machine learning was utilized to show that hippocampal radiomics signatures were strongly correlated with apolipoprotein E, cerebrospinal fluid β‐amyloid (Aβ), and cerebrospinal fluid tau.^19^ Moreover, at a five‐year follow‐up of MCI patients, hippocampal signatures were found to change with the longitudinal variation in cognitive performance measured by the Mini‐Mental State Examination (MMSE).^20^ In light of the robust and reproducible association of hippocampal radiomics signatures with AD, exploring the use of hippocampal radiomics in improving the accuracy of early diagnosis of AD is warranted.

PET/MRI offers the unique capability to simultaneously evaluate brain structure, CBF, and glucose metabolism with optimal spatial and temporal registration of both modalities. This methodology can aid in better understanding the impact on neuronal function and shed light on the mechanisms involved in the development of AD. In this study, we hypothesized that radiomics features extracted from the hippocampus imaging could provide insights into the underlying pathology of AD. Consequently, we aimed to develop and evaluate a machine learning model with hippocampal radiomics features extracted from PET/MRI for early diagnosis of AD.

## 2 MATERIALS AND METHODS

### 2.1 Participants

A total of 159 subjects were enrolled in this study from July 2017 to August 2022 at Xuanwu Hospital, Capital Medical University. The participants were predominantly right‐handed and included 53 HC participants, 55 aMCI patients, and 51 AD patients. The diagnosis of AD was based on the National Institute of Neurological and Communicative Diseases and Stroke/Alzheimer's Disease and Related Disorders Association criteria for probable.^21^ And the diagnosis of aMCI was based on diagnostic criteria defined by Petersen et al.^22^ The HC participants were age‐ and gender‐matched to AD patients and had no cognitive decline complaints, with the MMSE score ≥24. Those participants were excluded who had current or previous psychiatric or neurological disorders such as major depression, stroke, brain tumor, head injury, or cerebrovascular injury related to cognitive impairment.

This study was approved by the Ethics Committee of Xuanwu Hospital, Capital Medical University, China. All participants or their legal guardians gave written informed consent before the start of the study.

### 2.2 Imaging acquisition

All PET/MRI scans were performed within a 30‐day window following the neuropsychological assessment, including MMSE and Montreal Cognitive Assessment (MoCA), using a simultaneous time‐of‐flight (TOF) PET/MRI scanner (Signa, GE Healthcare, Waukesha, WI, USA). A 19‐channel head and neck union coil was used for imaging. Before scanning, all participants fasted for 4–6 h to ensure normal blood glucose levels. Each participant received an intravenous injection of 3.7 MBq/kg of ^18^F‐FDG while lying supine with their eyes closed in a dimly lit room.

MR images were acquired with the PET acquisition at the same time. High‐resolution T1‐weighted imaging (3D T1WI) were acquired using the following parameters: repetition time/echo time = 8.5/3.2 ms, flip angle = 15°, voxel size = 1.00 × 1.00 × 1.00 mm^3^, and 188 slices. Additionally, 3D ASL images were acquired using the following parameters: post‐labeling delay = 2.0 s,^23^ repetition time/echo time = 10.7/5337 ms, voxel size = 1.88 × 1.88 × 4.00 mm^3^, and 36 slices. Simultaneous PET data were acquired, using a 2‐point Dixon scan for MRI‐based PET attenuation correction. The PET data were reconstructed to a matrix of size 192 × 192, with a field of view of 35 cm and a slice thickness of 2.78 mm (each voxel measuring 1.82 × 1.82 × 2.78 mm^3^). This was achieved using an algorithm, incorporating TOF information, point spread function, and an ordered subset expectation maximization method. The algorithm involved 8 iterations, 32 subsets, and applied a 3 mm Gaussian filter.^12^

### 2.3 Data preprocessing

**FIGURE 1:**
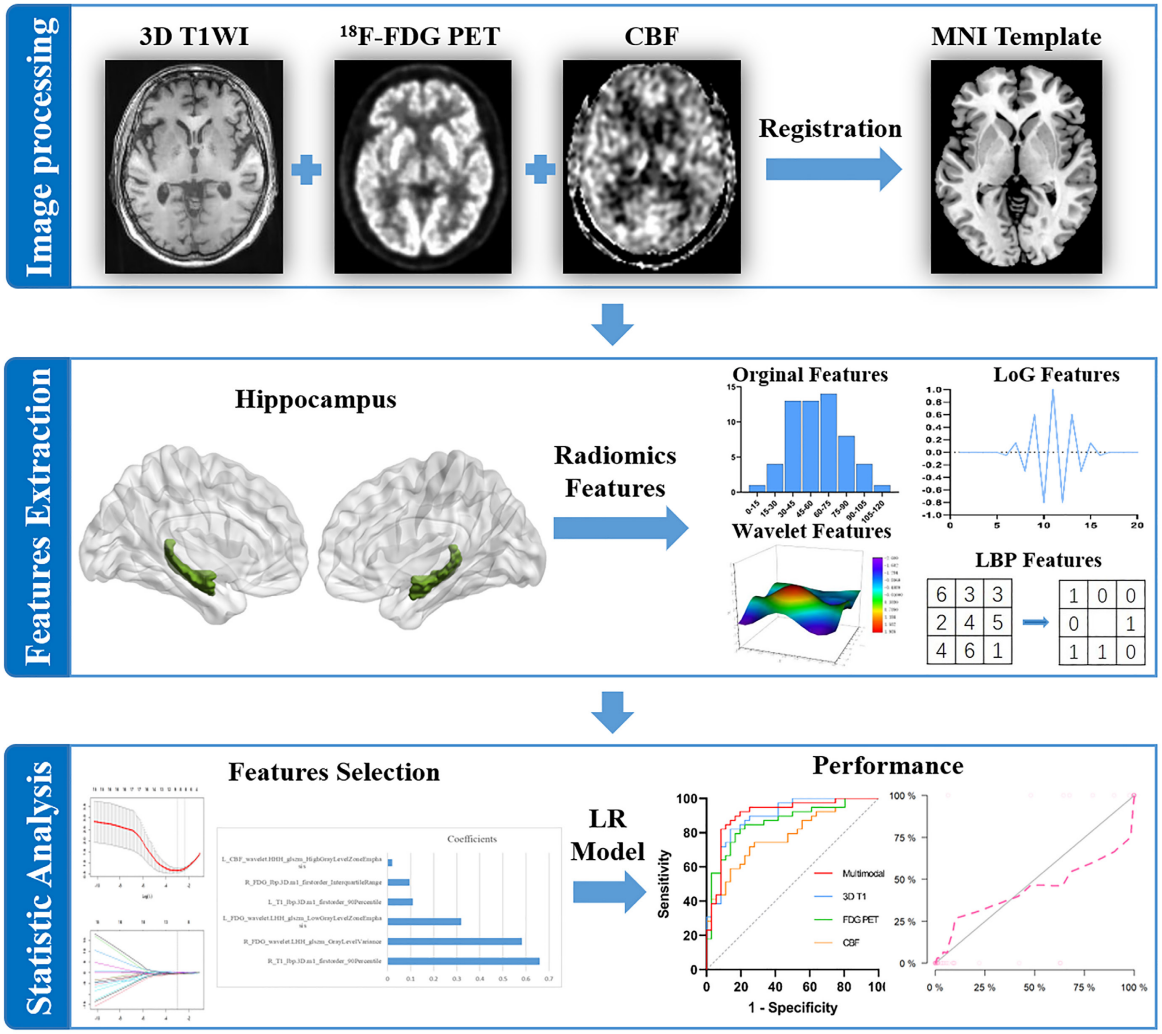
Radiomics workflow. 3D T1WI, 3 dimensional T1‐weighted imaging; ^18^F‐FDG PET, ^18^F‐fluorodeoxyglucose positron emission tomography; CBF, cerebral blood flow; MNI, Montreal Neurological Institute; Original features, including first‐order features, shape‐based features and texture features; LBP, local binary patterns; LoG, Laplacian of Gaussian; LR, logistic regression.

The diagram of this machine learning‐based analysis was overviewed in Figure 1.

PET and MRI images were processed using MATLAB 2018b (The MathWorks Inc.) and Statistical Parametric Mapping (SPM12, Wellcome Department of Imaging Neuroscience, London, United Kingdom). SPM cortical segmentation was used to divide the images into gray matter, white matter, and cerebrospinal fluid images. First, the ^18^F‐FDG PET and CBF images were aligned to the matched 3D T1WI images. The 3D T1WI images were then spatially normalized into the standard Montreal Neurological Institute (MNI) space. Subsequently, PET and CBF images were also normalized to the MNI space with the same transformation matrix. Moreover, ^18^F‐FDG PET images were converted to standardized uptake value ratio (SUVR) maps, referring to the pons.^24^, ^25^

### 2.4 Hippocampal segmentation and feature extraction

The bilateral hippocampal segmentation was performed using an MRI template, which was proposed by the Johns Hopkins Department of Radiology and drawn in standard MNI space manually by PMOD software (PMOD 4.002, Technologies Ltd., Zürich, Switzerland).^26^ The hippocampal volume, mean CBF, and mean SUVR of each subject were calculated for region of interest (ROI)‐wise analysis. For each modality, 1316 features were extracted respectively within the left and right hippocampus, including 18 first‐order features, 14 shape‐based features, 75 texture features, 186 Laplacian of Gaussian (LoG) features, 744 wavelet features, and 279 local binary patterns (LBP) features. The quantitative radiomics features were calculated using the PyRadiomics package which was implemented in Python 3.8.1.

### 2.5 Feature selection and radiomics model

For feature selection, two feature selection methods, Max‐Relevance and Min‐Redundancy (mRMR) and Least Absolute Shrinkage and Selection Operator (LASSO) were employed. Initially, mRMR was utilized to filter the redundant and irrelevant features. Then LASSO was employed to select the optimized subset of features by choosing the regular parameter λ and determining the number of the feature. Once the number of features was determined, the most predictive subset of features was selected and the corresponding coefficients were evaluated.

The logistic regression (LR) models were trained to classify AD from HC, AD from aMCI, aMCI from HC with five‐fold cross‐validation. The data of all subjects were randomized into training groups and testing groups in a ratio of 7:3. Based on the features extracted from 3D T1WI, ^18^F‐FDG PET, and CBF images, the classifiers were divided into multiple categories: 3D T1WI classifier, ^18^F‐FDG PET classifier, CBF classifier, MRI classifier which consisted of 3D T1WI and CBF, and multimodal classifier which combined all categories. And the clinical classifier consisted of clinical information.

### 2.6 Statistical analysis

The statistical analysis was performed using R 3.6.1 software. The metrics of the area under the receiver operating characteristic curve (AUC) with 95% confidence interval (CI), accuracy (ACC), sensitivity (SEN), specificity (SPE), positive predictive value (PPV), and negative predictive value (NPV) were evaluated to assess the performance of the LR model. The radiomics score (Rad‐Score) for each participant was calculated by summing the selected features of LR models which were weighted by their coefficients.

Furthermore, the decision curve analysis was conducted to assess the clinical utility of the model, which was constructed from the net benefit within a threshold probability range in the testing group. The codes used in this study are available from the corresponding author upon request.

The differences between demographic characteristics and imaging features were compared using SPSS 26 software. Shapiro–Wilk test was used to evaluate the distribution of continuous variables. One‐way Analysis of Variance (ANOVA) was conducted for data with continuous variables and a normal distribution, while the Wilcoxon test was used for data without normal distribution. Additionally, Kruskal–Wallis test was used for comparison of more than two groups. The differences between any two groups were verified using post hoc comparisons. Categorical variables were compared using chi‐squared testing. The statistical significance was determined at a level of *p* < 0.05.

## 3 RESULTS

### 3.1 Demographic characteristics

A total of 159 subjects were evaluated in this study. Significant group differences were found in age (*p* = 0.002), MMSE and MoCA scores (*p* < 0.001). The aMCI group was older than HC group (*p* < 0.001). However, no significant differences were found in terms of gender (*p* = 0.995) and education (*p* = 0.264). There were significant differences for the hippocampal volume, ^18^F‐FDG PET SUVR value, and CBF value (all *p* < 0.01). Specifically, the hippocampal volume (*p* < 0.001) and ^18^F‐FDG PET SUVR value (*p* < 0.001) decreased in the progressive stages of HC, aMCI, and AD. The CBF value of HC and aMCI were similar (*p* = 0.966) and both were higher than AD (*p* = 0.001). The detailed information is listed in Table 1.

**TABLE 1:** Demographics and imaging features of participants.

Parameters	AD	aMCI	HC	*p* Value			
				Groups	AD vs. HC	AD vs. aMCI	aMCI vs. HC
No. of subjects	51	55	53				
Age (years)	64.90 ± 8.12	68.21 ± 8.55	62.04 ± 10.08	0.002^a^	0.105	0.059	<0.001
Gender (M/F)	19/32	21/34	20/33	0.995^b^			
Education (years)	10.98 ± 3.85	11.96 ± 3.59	11.43 ± 3.36	0.264^c^			
MMSE score	17.33 ± 7.22	26.44 ± 2.83	28.17 ± 1.89	<0.001^c^	<0.001	<0.001	0.020
MoCA score	12.54 ± 6.93	21.65 ± 3.86	25.53 ± 2.91	<0.001^c^	<0.001	<0.001	<0.001
Hippocampal volume (mm^3^)	719.84 ± 181.21	929.09 ± 172.56	1023.36 ± 123.45	<0.001^c^	<0.001	<0.001	0.029
SUVR value	0.79 ± 0.12	0.81 ± 0.10	0.89 ± 0.09	<0.001^a^	<0.001	0.020	<0.001
CBF value	41.98 ± 8.05	46.84 ± 6.55	46.78 ± 6.72	0.001^a^	0.001	0.001	0.966

*Note*: The data are presented as mean ± standard deviation; SUVR value and CBF value were calculated in the hippocampus.

Abbreviations: AD, Alzheimer's disease; aMCI, amnestic mild cognitive impairment; CBF, cerebral blood flow; F, female; HC, healthy control; M, male; MMSE, Mini‐Mental State Examination; MoCA, Montreal Cognitive Assessment; SUVR, standardized uptake value ratio.

^a^ One‐way ANOVA.

^b^ Chi‐square test.

^c^ Kruskal–Wallis test.

All participants of HC, aMCI, and AD were randomly divided 7 to 3 into a training group and a testing group (Table 2).

**TABLE 2:** Demographics of datasets in training and testing group.

Parameters	AD		aMCI		HC	
	Training	Testing	Training	Testing	Training	Testing
No. of subjects	36	15	39	16	38	15
Age (years)	63.83 ± 7.77	67.47 ± 8.65	68.61 ± 8.17	67.25 ± 9.61	61.18 ± 10.22	64.20 ± 9.73
Gender (M/F)	12/24	7/8	14/25	7/9	13/25	7/8
Education (years)	11.11 ± 4.00	10.58 ± 3.48	11.56 ± 3.77	12.93 ± 3.02	11.42 ± 3.48	11.47 ± 3.18
MMSE score	17.17 ± 7.14	17.73 ± 7.69	26.18 ± 2.86	27.06 ± 2.74	28.11 ± 1.87	28.33 ± 1.99
MoCA score	12.29 ± 6.50	13.13 ± 8.04	20.97 ± 4.06	23.25 ± 2.82	25.42 ± 2.91	25.80 ± 2.98

*Note*: The data are presented as mean ± standard deviation.

Abbreviations: AD, Alzheimer's disease; aMCI, amnestic mild cognitive impairment; F, female; HC, healthy control; M, male; MMSE, Mini‐Mental State Examination; MoCA, Montreal Cognitive Assessment.

### 3.2 Radiomics features and Rad‐Score

**FIGURE 2:**
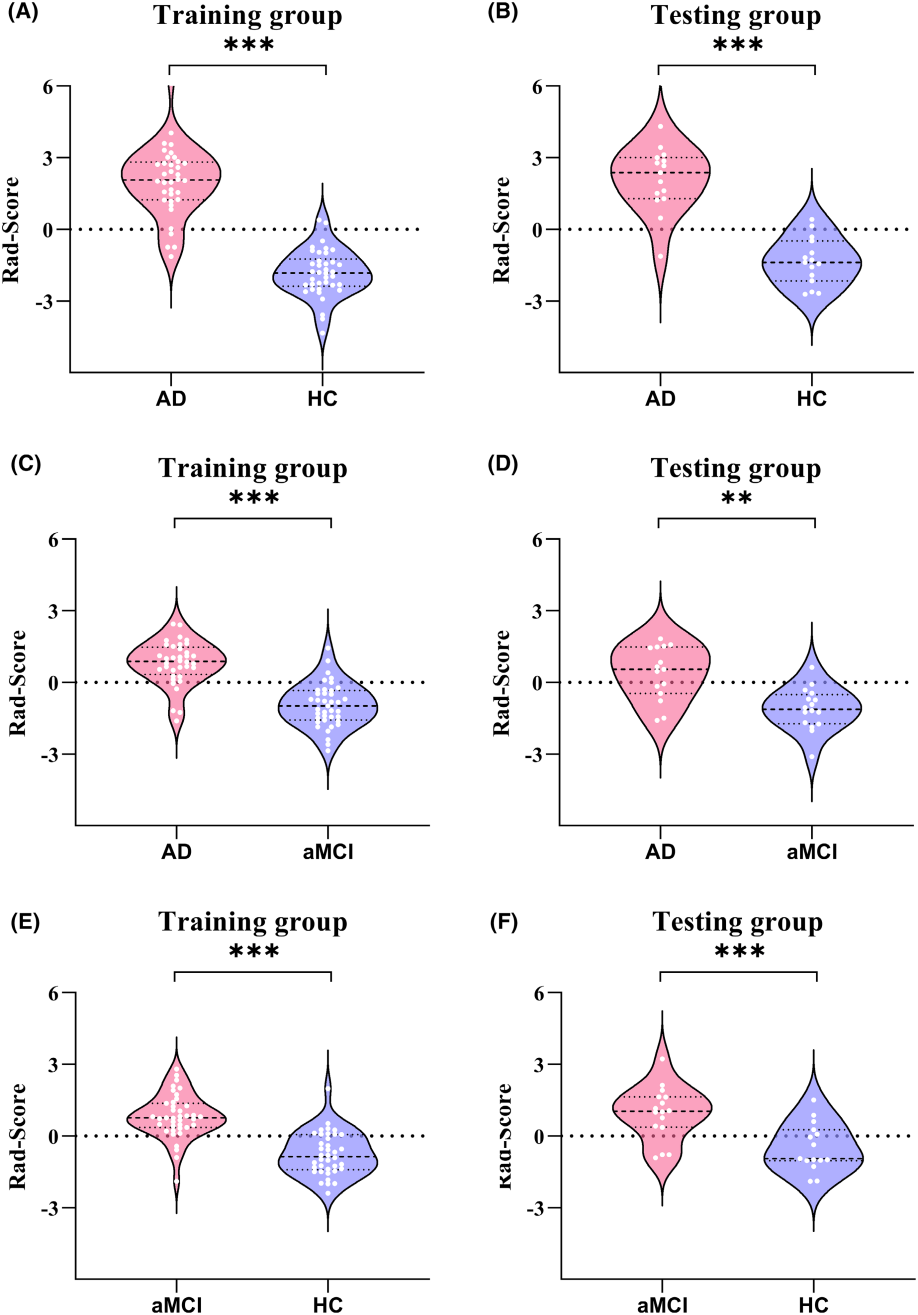
Rad‐Scores of the subjects in multimodal classifier. Rad‐Scores of multimodal classifier for AD and HC in training (A) and testing (B) groups; Rad‐Scores of multimodal classifier for AD and aMCI in training (C) and testing (D) groups; Rad‐Scores of multimodal classifier for aMCI and HC in training (E) and testing (F) groups. AD, Alzheimer's disease; aMCI, amnestic mild cognitive impairment; HC, healthy control; Rad‐Scores, radiomics scores. ***p < 0.001; **p < 0.01.

The comparisons of the Rad‐Score in multimodal classifier for classifying AD and HC, AD and aMCI, aMCI and HC showed significant differences (all *p* < 0.01) (Figure 2). For other classifiers with radiomics features, the Rad‐Scores were detailed in Data S1.

**FIGURE 3:**
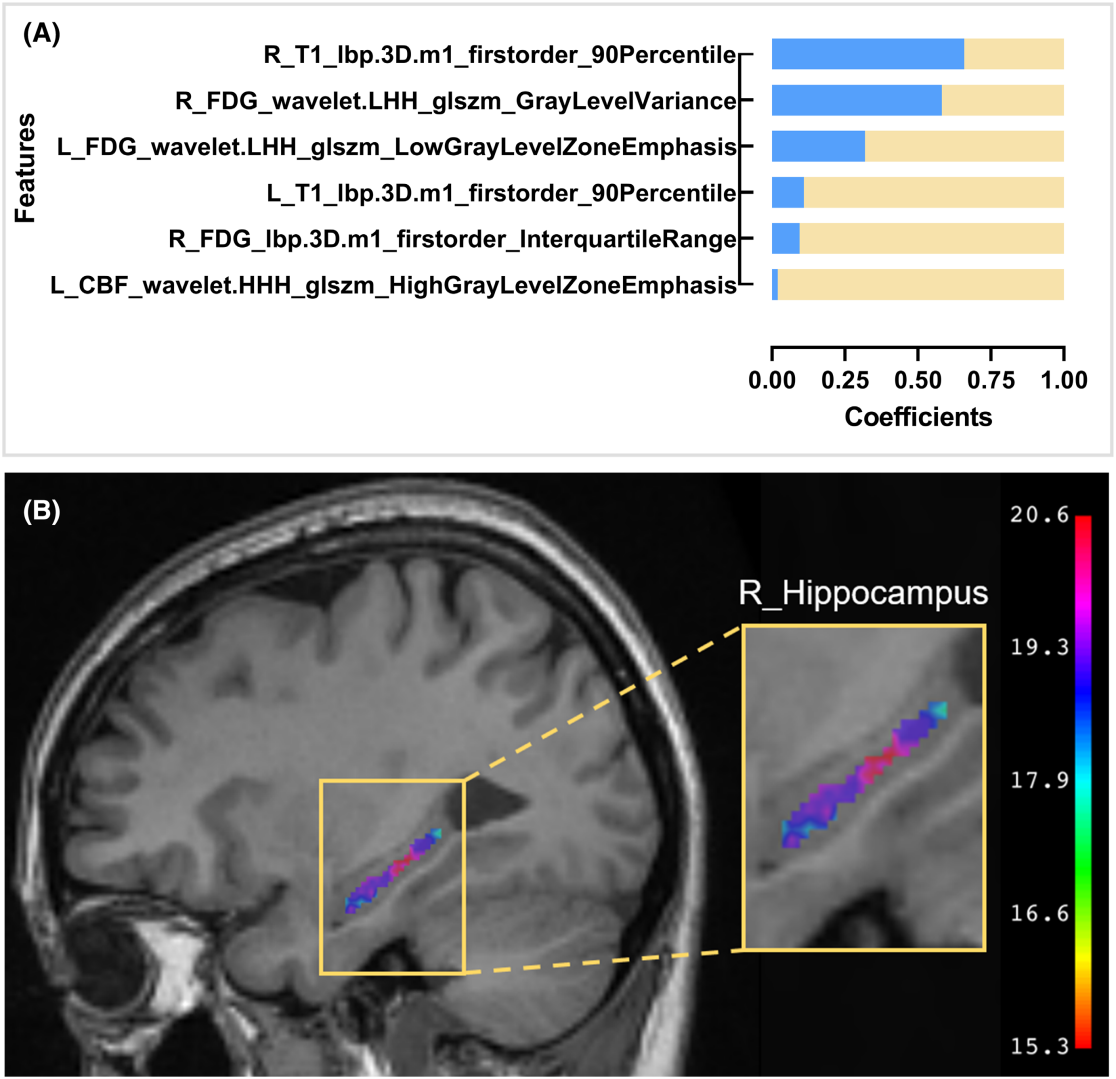
Radiomics features in multimodal classifier for identification of aMCI and HC. (A) the selected features and its coefficients in multimodal classifier for identification of aMCI and HC; (B) voxel‐wise level distribution of R_T1_lbp.3D.m1_firstorder_90Percentile. aMCI, amnestic mild cognitive impairment; glszm, gray level size zone matrix; HC, healthy control; lbp, local binary patterns; R, right.

In the multimodal classifier for distinguishing aMCI from HC, the categories and coefficients of the selected radiomics features were showed in Figure 3. Additionally, the Rad‐Score, which is the result of the linear combination of LR model, was calculated as below:

$$\begin{array}{c} \mathrm{Rad}‐\text{Score}\;=-0.020^{*}L\_\mathrm{CBF}\_\text{wavelet}.\mathrm{HHH}\_\text{glszm}\_\text{HighGrayLevelZoneEmphasis}+\\ \;-0.095^{*}R\_\mathrm{FDG}\_\mathrm{lbp}.3D.m1\_\text{firstorder}\_\text{InterquartileRange}+\\ \;-0.110^{*}L\_T1\_\mathrm{lbp}.3D.m1\_\text{firstorder}\_90\text{Percentile}+\\ \;-0.319^{*}L\_\mathrm{FDG}\_\text{wavelet}.\mathrm{LHH}\_\text{glszm}\_\text{LowGrayLevelZoneEmphasis}+\\ \;-0.582^{*}R\_\mathrm{FDG}\_\text{wavelet}.\mathrm{LHH}\_\text{glszm}\_\text{GrayLevelVariance}+\\ \;-0.659^{*}R\_T1\_\mathrm{lbp}.3D.m1\_\text{firstorder}\_90\text{Percentile}+0.038 \end{array}$$

### 3.3 Diagnostic performance

**FIGURE 4:**
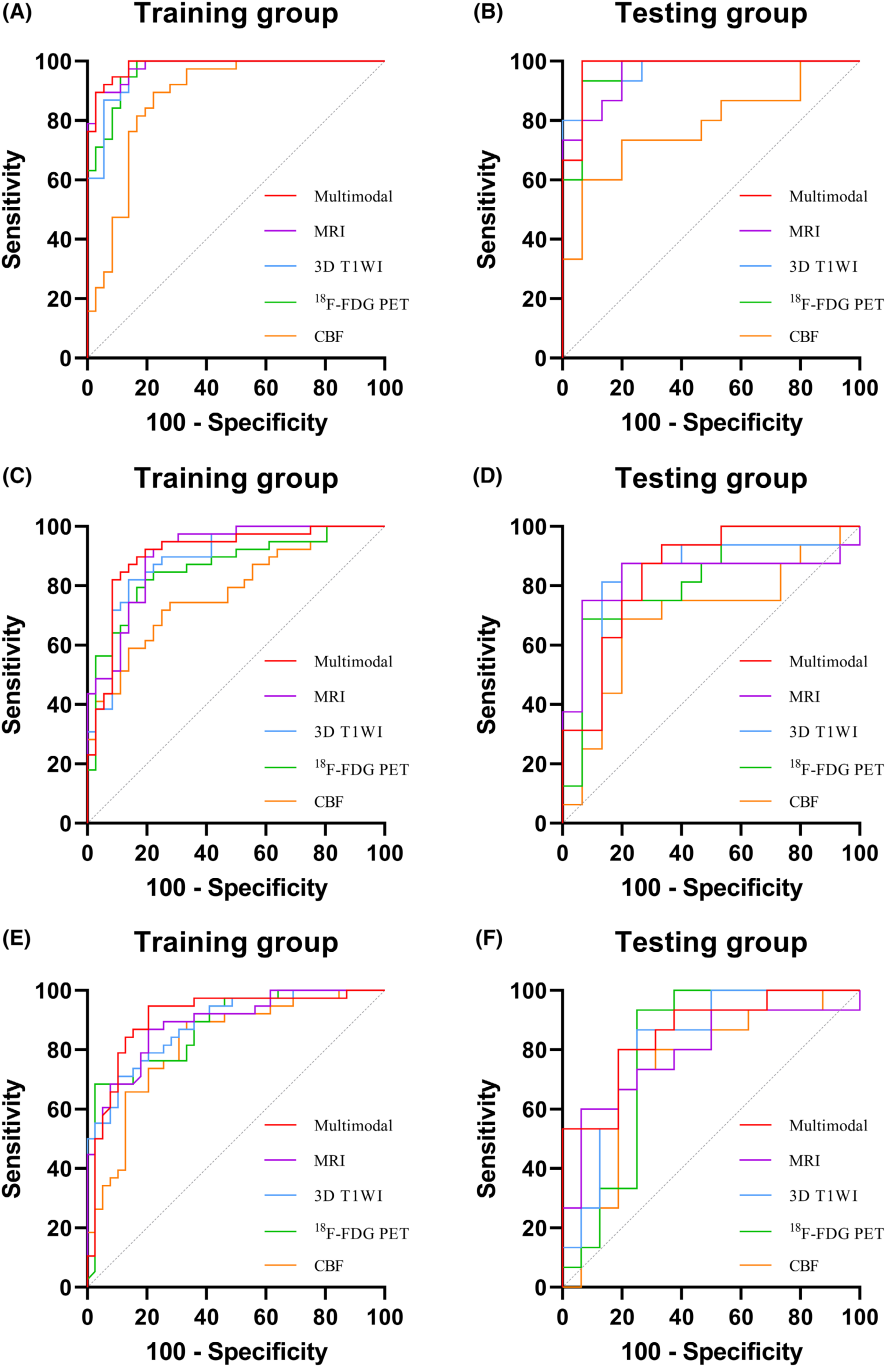
The ROC of classifiers. (A and B), ROC of classification for AD and HC; (C and D), ROC of classification for AD and aMCI; (E and F), ROC of classification for aMCI and HC; Multimodal, Multimodal (3D T1WI + ^18^F‐FDG PET + CBF) classifier; MRI, MRI (3D T1WI + CBF) classifier; ROC, receiver operating characteristic curve; AD, Alzheimer's disease; aMCI, amnestic mild cognitive impairment; HC, healthy control.

The performance of using single modal (3D T1WI, ^18^F‐FDG PET and CBF), MRI (3D T1WI + CBF) and multimodal (3D T1WI + ^18^F‐FDG PET + CBF) classifiers in the hippocampus were evaluated, as shown in Figure 4, Tables 3 and 4. We found that the multimodal classifier achieved better performance in discriminating AD, aMCI, and HC than single modals and MRI classifiers. In the classification of AD and HC, the multimodal classifier yielded AUC = 0.98 and ACC = 96.7% in the testing group, which is higher than the MRI classifier (AUC = 0.96, ACC = 90.0%) and the single‐level hippocampal features (3D T1WI, AUC = 0.96, ACC = 90.0%; ^18^F‐FDG PET, AUC = 0.96, ACC = 93.3%; CBF, AUC = 0.78, ACC = 76.7%), as shown in Figure 4A,B.

**TABLE 3:** Performance of single‐modal machine learning models.

	AD vs. HC			AD vs. aMCI			aMCI vs. HC		
	3D T1WI	^18^F‐FDG PET	CBF	3D T1WI	^18^F‐FDG PET	CBF	3D T1WI	^18^F‐FDG PET	CBF
Training									
AUC	0.97	0.97	0.88	0.89	0.86	0.78	0.89	0.87	0.82
95% CI	0.93–1.00	0.93–1.00	0.79–0.96	0.82–0.97	0.78–0.95	0.68–0.88	0.82–0.96	0.80–0.95	0.73–0.92
ACC	91.9%	91.9%	83.8%	84.0%	81.3%	73.3%	80.5%	83.1%	77.9%
SEN	97.4%	94.7%	89.5%	82.1%	79.5%	71.8%	71.1%	68.4%	89.5%
SPE	86.1%	88.9%	77.8%	86.1%	83.3%	75.0%	89.7%	97.4%	66.7%
PPV	88.1%	90.0%	81.0%	86.5%	83.8%	75.7%	87.1%	96.3%	72.3%
NPV	96.9%	94.1%	78.6%	81.6%	78.9%	71.1%	76.1%	76.0%	86.7%
Testing									
AUC	0.96	0.96	0.78	0.83	0.83	0.69	0.83	0.80	0.73
95% CI	0.90–1.00	0.90–1.00	0.61–0.95	0.67–1.00	0.68–0.98	0.49–0.89	0.67–0.98	0.62–0.97	0.54–0.92
ACC	90.0%	93.3%	76.7%	80.9%	80.6%	74.2%	80.6%	83.8%	74.2%
SEN	80.0%	93.3%	73.3%	81.3%	68.8%	68.8%	86.7%	93.3%	80.0%
SPE	100%	93.3%	80.0%	86.7%	93.3%	80.0%	75.0%	75.0%	68.8%
PPV	100%	93.3%	78.6%	86.7%	91.7%	78.6%	76.5%	77.8%	70.6%
NPV	83.3%	93.3%	75.0%	81.3%	73.7%	70.6%	85.7%	92.3%	78.6%

Abbreviations: AD, Alzheimer's disease; aMCI, amnestic mild cognitive impairment; AUC, the area under the receiver operating characteristic curve; CI, confidence interval; ACC, accuracy; HC, healthy control; NPV, negative predictive value; PPV, positive predictive value; SEN, sensitivity; SPE, specificity.

**TABLE 4:** Performance of combination models.

	AD vs. HC		AD vs. aMCI		aMCI vs. HC	
	Multimodal	MRI	Multimodal	MRI	Multimodal	MRI
Training						
AUC	0.99	0.98	0.91	0.91	0.91	0.89
95% CI	0.97–1.00	0.96–1.00	0.83–0.98	0.84–0.97	0.84–0.98	0.82–0.96
ACC	93.2%	93.2%	86.7%	85.30%	87.0%	83.1%
SEN	89.5%	89.5%	82.1%	89.70%	94.7%	86.8%
SPE	97.2%	97.2%	91.7%	80.60%	79.5%	79.5%
PPV	97.1%	97.1%	91.4%	83.30%	81.8%	80.5%
NPV	89.7%	89.7%	82.5%	87.90%	93.9%	86.1%
Testing						
AUC	0.98	0.96	0.85	0.83	0.86	0.79
95% CI	0.93–1.00	0.90–1.00	0.70–0.99	0.66–1.00	0.73–0.99	0.62–0.96
ACC	96.7%	90.0%	80.6%	83.9%	80.6%	77.4%
SEN	100%	100%	87.5%	75.0%	80.0%	60.0%
SPE	93.3%	80.0%	73.3%	93.3%	81.3%	93.8%
PPV	93.8%	83.3%	77.8%	92.3%	80.0%	90.0%
NPV	100%	100%	84.6%	77.8%	81.3%	71.4%

Abbreviations: ACC, accuracy; AD, Alzheimer's disease; aMCI, amnestic mild cognitive impairment; AUC, the area under the receiver operating characteristic curve; CI, confidence interval; HC, healthy control; Multimodal, Multimodal (3D T1WI + ^18^F‐FDG PET + CBF) classifier; MRI, MRI (3D T1WI + CBF) classifier; NPV, negative predictive value; PPV, positive predictive value; SEN, sensitivity; SPE, specificity.

Regarding the classification of aMCI and HC, using the comprehensive characterization of hippocampal feature ensemble resulted in an AUC = 0.86 and ACC = 80.6% in the testing group, and the MRI classifier had an AUC = 0.79 and ACC = 77.4% (Figure 4E,F).

Furthermore, the performance of the multimodal classifier was evaluated in female and male subgroups with AUC = 0.85 and ACC = 83.6% for the female subgroup and AUC = 0.94 and ACC = 90.2% for the male subgroup in the classification of aMCI and HC (Data S2, Table S1).

### 3.4 Clinical classifier and application for aMCI

**FIGURE 5:**
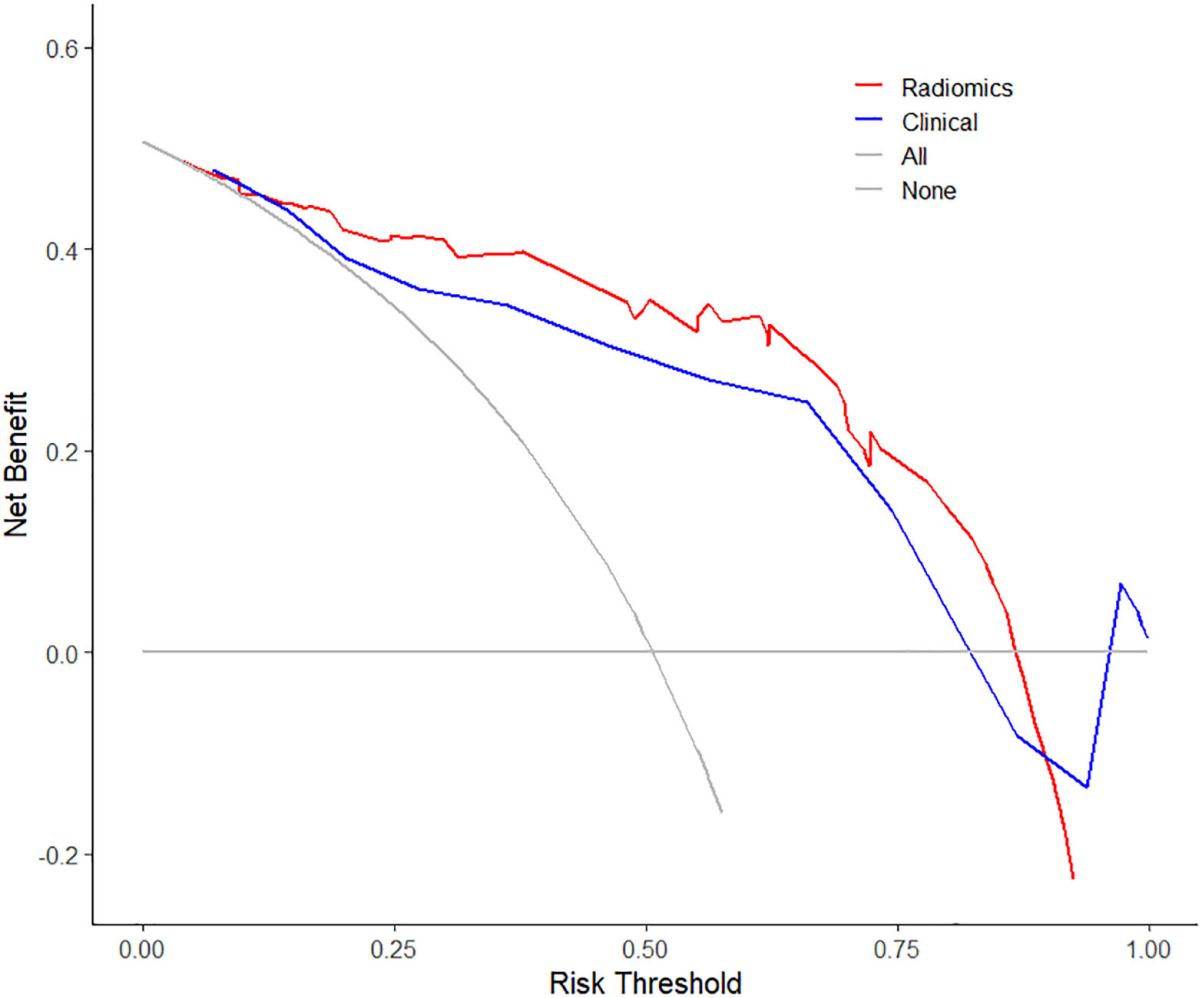
Decision curve analysis of Radiomics model and Clinical model in the testing group for aMCI patients and HC participants. The *Y*‐axis means the model benefit, and the *X*‐axis represents the threshold probability. Radiomics, including multimodal features extracted from 3D T1WI, ^18^F‐FDG PET, and ASL‐CBF of aMCI and HC; Clinical, including MoCA scores of aMCI and HC. aMCI, amnestic mild cognitive impairment; HC, healthy control; MoCA, Montreal Cognitive Assessment.

Both univariate logistic regression and multivariate logistic regression were conducted for gender, age, education, MMSE, and MoCA. The results showed that MoCA was *p* < 0.001, and the AUC of MoCA to identify aMCI from HC was 0.82 (95% CI: 0.73–0.92) in the training group, 0.75 (95% CI: 0.57–0.93) in the testing group. The decision curve analysis indicated a threshold probability between 0 and 0.85 to benefit from the Radiomics model and was higher than the Clinical model in the range of 0.1 to 0.85, as shown in Figure 5.

## 4 DISCUSSION

In this study, we developed LR models by utilizing single‐level hippocampal radiomics features derived from 3D T1WI, ^18^F‐FDG PET, and CBF respectively, to classify AD, aMCI, and HC. The hippocampal multi‐features ensemble based on structural, metabolic, and blood flow information in the multimodal classifier improved the accuracy of AD and aMCI identification.

Hippocampal atrophy is widely observed in AD and aMCI patients and is strongly correlated with disease progression. A five‐year follow‐up study has consistently shown a relationship between reduced hippocampal volume and increased risk of AD.^27^ Our research supports these findings, that is, the hippocampal volume differs significantly among HC, aMCI, and AD, and exhibits a trend toward worsening atrophy with disease exacerbation. The same results could also be concluded from ^18^F‐FDG PET SUVR of the hippocampus in this study. This is consistent with the study of Laforce et al.^28^ in which they reported that decreased glucose metabolism in the hippocampus preceded volume atrophy and was also a potential biomarker for AD. For patients with AD and aMCI, the voxel‐wise and ROI‐based group differences in a simultaneous PET/MRI study demonstrated that the pattern of abnormal CBF was similar to the hypometabolism pattern of ^18^F‐FDG PET.^29^ Similarly, another simultaneous PET/MRI study suggested CBF had comparable efficacy to ^18^F‐FDG PET for AD classification.^30^ Our study also showed that blood perfusion in the hippocampus was significantly reduced in patients with AD, whereas not in patients with aMCI when compared to the HC group. This indicated that CBF can be effectively used as a biomarker for identifying AD from HC, but is insufficient for identifying aMCI from HC.

One of the challenges for modern neuroimaging is the early diagnosis of AD, particularly for patients with aMCI, because the current AD therapeutics were developed to focus on early‐stage AD for optimal efficacy.^31^, ^32^, ^33^ Machine learning, including its offshoot deep learning, has emerged as a highly promising technique for disease diagnosis.^34^, ^35^, ^36^ Numerous studies have exploited machine learning to assist imaging analysis in the classification of HC, MCI, and AD.^37^, ^38^, ^39^ In contrast to the random forest models based on the whole brain area analysis of Huang et al.^40^ (AUC: 0.99 and ACC: 93.5% for AD; AUC: 0.88 and ACC: 80.8% for MCI), our hippocampal radiomics classifiers (AUC: 0.98 and ACC: 96.7% for AD; AUC: 0.86 and ACC: 80.6% for aMCI) outperformed comparably in discriminating AD and aMCI from HC. The single brain region analysis in our study evidently minimized the instability variables related to multiple brain regions, simplified the training, and enhanced the robustness of machine learning.

In this study, the hippocampus was defined as ROI for single brain region analysis, and the radiomics approach was employed for texture analysis of PET/MRI data for efficient performance. Hippocampal volume can only coarsely represent complex anatomical atrophy in AD. A study of the hippocampus suggested that hippocampal anatomical heterogeneity determined AD‐induced heterogeneous microscopic changes within the hippocampus.^41^ Differential changes within the hippocampal tissue are reflected as altered texture patterns in medical images, which can be quantitatively measured by radiomics features. Previous studies by Zhao et al.,^19^ Feng et al.,^18^ and Du et al.^42^ developed a 3D T1WI‐based hippocampal radiomics classifier to diagnose AD. These studies reported AUCs between 0.92 and 0.95, ACCs between 86.8% and 88.2% for the classification of AD and HC, and AUCs between 0.69 and 0.70, ACCs between 67.8% and 70.5% for the classification of MCI and HC. In comparison, our multimodal classifier significantly improved the diagnostic performance of AD (AUC = 0.98, ACC = 96.7%), especially in aMCI (AUC = 0.86, ACC = 80.6%). Zhao et al.^19^ found a significant correlation (up to r = 0.40, *p* < 0.001) between texture features, represented by gray level size zone matrix features, and MMSE. Consistent with this, the gray level size zone matrix features were an important component of the selected features in this study. This may indicate that radiomics does have the potential to reflect robust and reproducible changes in hippocampal pathological alterations in AD and aMCI. Furthermore, the clinical benefit of our multimodal classifier outperformed the MoCA and had the potential to replace the clinical cognitive function assessment scale to simplify tedious diagnostic work. The significant advantage of our study lies in the utilization of ^18^F‐FDG PET and CBF to complement 3D T1WI by providing metabolic and blood flow information. As a result, this allows for better identification of aMCI for early diagnosis. A recent study has found that ^18^F‐FDG hypometabolism in the hippocampus differs among subregions in AD.^12^ ^18^F‐FDG PET‐based hippocampal radiomics can also be applied to delineate hippocampal heterogeneity to identify AD and aMCI with an efficacy close to 3D T1WI. However, there are few studies on CBF‐based hippocampal subregions. This is mainly because the CBF does not have good enough resolution for fine brain regions, which explains why the efficacy of the CBF classifier in our study is not as superior as other classifiers. We further constructed an MRI model and found it to be effective in AD classification. However, the multimodal hippocampal parameters ensemble had better performance in the classification of aMCI and HC. This suggests that the metabolic information provided by ^18^F‐FDG PET can be complementary to MRI.

Overall, classifiers composed of single‐level hippocampal features were effective in diagnosing AD or aMCI from HC, and the multi‐feature ensemble classifier performed best, especially in aMCI, and had favorable clinical benefits.

However, our study had several limitations that we need to address. Firstly, this study was conducted in a single center and only recruited patients from our hospital. To enhance the efficacy and robustness of the machine learning model in this study, a multi‐center study could increase the size of the data and validate the generalization ability of the model. In the future, the results of this study will be evaluated on multi‐centers data. Secondly, the hippocampal segmentation utilized in this study was atlas‐based and was subject to individual differences. An accurate automatic segmentation that can be performed in the individual space will be a valuable research perspective. Lastly, we did not analyze Aβ PET in this study, which is available for risk stratification of patients with early AD.^43^ We are in the process of acquiring Aβ PET data and the results of amyloid PET data will be evaluated further in the near future at our PET center and other larger cohort studies.

## 5 CONCLUSION

In this study, we developed a novel approach based on ^18^F‐FDG PET, ASL‐CBF, and 3D T1WI for early diagnosis of AD with hippocampal radiomics using multiparametric PET/MRI. Our findings revealed that radiomics features can reflect hippocampal structural and metabolic impairments, where MRI and ^18^F‐FDG PET are of equal value in AD. 3D T1WI is superior to ^18^F‐FDG PET and CBF in aMCI, and multiparametric hippocampal signatures of these ensembles could further improve diagnostic efficacy.

## AUTHOR CONTRIBUTIONS

All authors contributed to the study's conception and design. The study was designed by Shaozhen Yan and Jie Lu. Material preparation, data collection, and analysis were performed by Zhigeng Chen, Sheng Bi, Bixiao Cui, Yi Shan, Hongwei Yang, Zhigang Qi, Zhilian Zhao, and Ying Han. The first draft of the manuscript was written by Zhigeng Chen and all authors commented on previous versions of the manuscript. All authors read and approved the final manuscript.

## FUNDING INFORMATION

This study was supported by the National Natural Science Foundation of China (Grant No. 82102010), Beijing Nova Program (Grant No. Z211100002121054, 20220484177), Beijing Brain Initiative from Beijing Municipal Science & Technology Commission (Grant No. Z201100005520018).

## CONFLICT OF INTEREST STATEMENT

The authors declare no conflicts of interest.

## Supporting information


Data S1.

Data S2.

## Data Availability

The data that support the findings of this study are available from the corresponding author upon reasonable request.
