# Supplementary material for: Multiparametric hippocampal signatures for early diagnosis of Alzheimer's disease using 18F‐FDG PET/MRI Radiomics
Source: CNS Neurosci Ther. 2023 Nov 30;30(4):e14539. doi: 10.1111/cns.14539 (PMC11017421; doi:10.1111/cns.14539)
Supplement: Supplementary file 1 — Data S1. [file CNS-30-e14539-s002.docx]

**Multiparametric Hippocampal Signatures for Early Diagnosis of Alzheimer's Disease using ^18^F-FDG PET/MRI Radiomics**

**Outline**

[**Outline** 1](#_Toc138112054)

[**Multimodal classifier** 2](#_Toc138112054)

[**AD and HC** 2](#_Toc138112054)

[**AD and aMCI** 2](#_Toc138112054)

[**MRI classifier** 3](#_Toc138112054)

[**AD and HC** 3](#_Toc138112054)

[**AD and aMCI** 3](#_Toc138112054)

[**aMCI and HC** 3](#_Toc138112054)

[**3D T1WI classifier** 6](#_Toc138112054)

[**AD and HC** 6](#_Toc138112054)

[**AD and aMCI** 6](#_Toc138112054)

[**aMCI and HC** 6](#_Toc138112054)

[**^18^F-FDG PET classifier** 9](#_Toc138112054)

[**AD and HC** 9](#_Toc138112054)

[**AD and aMCI** 9](#_Toc138112054)

[**aMCI and HC** 9](#_Toc138112054)

[**CBF classifier** 12](#_Toc138112054)

[**AD and HC** 12](#_Toc138112054)

[**AD and aMCI** 12](#_Toc138112054)

[**aMCI and HC** 12](#_Toc138112054)

**Multimodal classifier**

The radiomics score (Rad-Score) of multimodal classifier for identifying Alzheimer’s disease (AD) and health control (HC), AD and amnestic mild cognitive impairment (aMCI), which is the result of the linear combination of Logistic regression (LR) model, was calculated as below:

**AD and HC**

Rad-Score =

-0.147*L_CBF_wavelet.HHH_glszm_HighGrayLevelZoneEmphasis+

-0.485*R_T1_log.sigma.2.0.mm.3D_glszm_GrayLevelNonUniformity+

-0.463*L_T1_lbp.3D.m1_firstorder_InterquartileRange+

0.365*R_FDG_wavelet.LHL_firstorder_Mean+

0.347*L_FDG_wavelet.LLH_firstorder_Mean+

-0.768*R_T1_log.sigma.3.0.mm.3D_gldm_LargeDependenceHighGrayLevelEmphasis+

-0.069*R_T1_lbp.3D.m1_firstorder_90Percentile+

-0.491*L_FDG_log.sigma.3.0.mm.3D_glcm_MaximumProbability+ 0.036

**AD and aMCI**

Rad-Score =

-0.038*L_CBF_log.sigma.2.0.mm.3D_glszm_SizeZoneNonUniformity+

0.413*L_T1_wavelet.HLH_firstorder_InterquartileRange+

-0.039*L_FDG_log.sigma.3.0.mm.3D_glszm_SizeZoneNonUniformity+

-0.228*R_T1_wavelet.LLL_glszm_ZoneVariance+

-0.431*R_T1_lbp.3D.k_ngtdm_Busyness+

-0.477*L_T1_wavelet.LHH_ngtdm_Coarseness+

0.168*R_FDG_lbp.3D.m1_firstorder_TotalEnergy + -0.112

**MRI classifier**

The comparison of the Rad-Score in MRI classifier for identifying AD and HC, AD and aMCI, aMCI and HC were presented in **Fig. S1**.

The Rad-Score, which is the result of the linear combination of LR model, was calculated as below:

**AD and HC**

Rad-Score =

-0.112*R_T1_log.sigma.2.0.mm.3D_glszm_GrayLevelNonUniformity+

-0.507*L_CBF_wavelet.HHH_glszm_ZoneEntropy+

-0.079*L_T1_lbp.3D.m1_firstorder_InterquartileRange+

-0.42*L_CBF_wavelet.HHH_glszm_HighGrayLevelZoneEmphasis+

-0.312*R_CBF_wavelet.HHL_glszm_SizeZoneNonUniformity+

0.914*L_T1_wavelet.LLH_glszm_ZoneEntropy+

0.088*R_T1_wavelet.LHL_gldm_DependenceEntropy+

-1.846*L_T1_log.sigma.3.0.mm.3D_firstorder_Kurtosis+

-1.991*R_T1_lbp.3D.m1_firstorder_InterquartileRange+

-0.279*R_T1_wavelet.LLL_ngtdm_Busyness+

-0.301*R_T1_wavelet.LLL_glszm_GrayLevelNonUniformity + 0.188

**AD and aMCI**

Rad-Score =

-0.251*R_CBF_wavelet.HHL_glszm_SizeZoneNonUniformity+

-0.251*L_T1_wavelet.HHL_glcm_MaximumProbability+

0.604*L_T1_wavelet.HHL_firstorder_Maximum+

0.463*R_T1_wavelet.LLH_glszm_SizeZoneNonUniformityNormalized+

-0.518*R_T1_lbp.3D.k_ngtdm_Busyness+

-0.044*R_CBF_wavelet.HLH_glszm_GrayLevelNonUniformityNormalized+

0.123*L_T1_wavelet.HLH_firstorder_InterquartileRange + -0.145

**aMCI and HC**

Rad-Score =

-0.1*R_CBF_wavelet.HHH_glszm_GrayLevelVariance+

-0.315*R_T1_lbp.3D.m1_firstorder_90Percentile+

-0.074*L_T1_lbp.3D.m1_firstorder_Median+

0.63*L_T1_wavelet.LHL_glcm_Correlation+

-0.449*L_CBF_wavelet.HLL_glszm_ZonePercentage+

-0.386*R_T1_original_glcm_ClusterShade + 0.097

**

**

Figure S1. Rad-Scores of the subjects in MRI classifier. **a**, Rad-Scores of AD and HC in training group; **b**, Rad-Scores of AD and HC in testing group; **c**, Rad-Scores of AD and aMCI in training group; **d**, Rad-Scores of AD and aMCI in testing group; **e**, Rad-Scores of aMCI and HC in training group; **f**, Rad-Scores of aMCI and HC in testing group; Rad-Scores, radiomics scores; MRI, MRI (3D T1WI + CBF) classifier; AD, Alzheimer’s disease; aMCI, amnestic mild cognitive impairment; HC, health control; *******, *P* < 0.001; ******, *P* < 0.01.

**3D T1WI classifier**

The comparisons of the Rad-Score in 3D T1WI classifier for identifying AD and HC, AD and aMCI, aMCI and HC were presented in **Fig. S2**.

The Rad-Score, which is the result of the linear combination of LR model, was calculated as below:

**AD and HC**

Rad-Score =

-0.701*L_T1_lbp.3D.m1_firstorder_InterquartileRange+

-0.48*R_T1_wavelet.LLL_glszm_GrayLevelNonUniformity+

-0.31*R_T1_log.sigma.2.0.mm.3D_glszm_GrayLevelNonUniformity+

-0.529*L_T1_log.sigma.3.0.mm.3D_firstorder_Kurtosis+

0.32*L_T1_log.sigma.2.0.mm.3D_glcm_Imc2+

0.199*R_T1_wavelet.LLH_glcm_ClusterShade+

-0.089*R_T1_lbp.3D.m1_firstorder_90Percentile+

-0.055*L_T1_log.sigma.2.0.mm.3D_gldm_DependenceVariance + 0.001

**AD and aMCI**

Rad-Score =

-0.27*L_T1_log.sigma.2.0.mm.3D_glszm_ZoneVariance+

0.003*L_T1_wavelet.HLH_ngtdm_Complexity+

-0.235*L_T1_wavelet.LHH_ngtdm_Coarseness+

-0.362*R_T1_lbp.3D.k_ngtdm_Busyness+

0.139*L_T1_log.sigma.2.0.mm.3D_glszm_SmallAreaEmphasis+

0.547*L_T1_wavelet.HLH_firstorder_InterquartileRange+

0.205*R_T1_wavelet.HLL_glcm_Correlation+

0.044*L_T1_wavelet.LLH_firstorder_InterquartileRange+

-0.065*L_T1_lbp.3D.k_firstorder_Entropy + -0.111

**aMCI and HC**

Rad-Score =

-0.635*R_T1_lbp.3D.m1_firstorder_90Percentile+

-0.415*R_T1_original_firstorder_Skewness+

-0.264*L_T1_log.sigma.3.0.mm.3D_firstorder_Kurtosis+

-0.101*R_T1_lbp.3D.m2_firstorder_90Percentile+

0.015*L_T1_wavelet.LHH_ngtdm_Coarseness+

-0.138*R_T1_wavelet.HLH_glcm_ClusterShade+

-0.164*R_T1_log.sigma.3.0.mm.3D_firstorder_Kurtosis+

-0.266*R_T1_lbp.3D.m2_firstorder_InterquartileRange+

-0.016*L_T1_lbp.3D.m2_firstorder_90Percentile+

0.096*R_T1_wavelet.LLL_gldm_LargeDependenceHighGrayLevelEmphasis + 0.03

**

**

Figure S2. Rad-Scores of the subjects in 3D T1WI classifier. **a**, Rad-Scores of AD and HC in training group; **b**, Rad-Scores of AD and HC in testing group; **c**, Rad-Scores of AD and aMCI in training group; **d**, Rad-Scores of AD and aMCI in testing group; **e**, Rad-Scores of aMCI and HC in training group; **f**, Rad-Scores of aMCI and HC in testing group; Rad-Scores, radiomics scores; AD, Alzheimer’s disease; aMCI, amnestic mild cognitive impairment; HC, health control; *******, *P* < 0.001; ******, *P* < 0.01.

**^18^F-FDG PET classifier**

The comparison of the Rad-Score in ^18^F-FDG PET classifier for identifying AD and HC, AD and aMCI, aMCI and HC were presented in Fig. S3.

The Rad-Score, which is the result of the linear combination of LR model, was calculated as below:

**AD and HC**

Rad-Score =

-0.638*L_FDG_wavelet.LLH_glcm_ClusterShade+

0.784*R_FDG_lbp.3D.m1_firstorder_Mean+

-0.245*L_FDG_log.sigma.3.0.mm.3D_glcm_MaximumProbability+

-0.399*R_FDG_wavelet.LHH_glszm_GrayLevelVariance+

0.136*R_FDG_wavelet.LHL_firstorder_Median+

-0.368*R_FDG_wavelet.LLH_glrlm_LongRunLowGrayLevelEmphasis+

0.186*R_FDG_wavelet.LLH_firstorder_Mean + 0.012

**AD and aMCI**

Rad-Score =

-0.179*R_FDG_wavelet.HHH_glszm_HighGrayLevelZoneEmphasis+

-0.398*L_FDG_wavelet.HHL_glszm_GrayLevelNonUniformityNormalized+

0.241*R_FDG_lbp.3D.m1_firstorder_Skewness+

-0.157*L_FDG_wavelet.LHL_glszm_SizeZoneNonUniformity+

0.217*L_FDG_lbp.3D.m2_ngtdm_Busyness+

0.659*L_FDG_wavelet.HHH_gldm_SmallDependenceLowGrayLevelEmphasis+

0.308*L_FDG_log.sigma.3.0.mm.3D_glcm_Imc2+

-0.076*L_FDG_wavelet.HHH_glszm_HighGrayLevelZoneEmphasis+

-0.374*L_FDG_wavelet.HHL_glszm_ZonePercentage+

-0.264*R_FDG_wavelet.HHL_glszm_LowGrayLevelZoneEmphasis+

-0.046*R_FDG_wavelet.LHL_glszm_ZonePercentage+

0.29*R_FDG_lbp.3D.m1_firstorder_TotalEnergy + -0.089

**aMCI and HC**

Rad-Score =

-0.267*R_FDG_original_firstorder_Minimum+

0.276*L_FDG_wavelet.LHL_firstorder_Median+

0.06*R_FDG_lbp.3D.m1_glcm_Correlation+

-0.209*R_FDG_wavelet.LHH_glszm_GrayLevelVariance+

-0.125*L_FDG_wavelet.LHH_glszm_LowGrayLevelZoneEmphasis+

0.081*L_FDG_wavelet.HLH_glszm_SizeZoneNonUniformity+

-0.112*L_FDG_original_firstorder_10Percentile+

-0.164*R_FDG_original_firstorder_10Percentile+

-0.232*L_FDG_log.sigma.3.0.mm.3D_glcm_MaximumProbability + 0.063

**

**

Figure S3. Rad-Scores of the subjects in ^18^F-FDG PET classifier. **a**, Rad-Scores of AD and HC in training group; **b**, Rad-Scores of AD and HC in testing group; **c**, Rad-Scores of AD and aMCI in training group; **d**, Rad-Scores of AD and aMCI in testing group; **e**, Rad-Scores of aMCI and HC in training group; **f**, Rad-Scores of aMCI and HC in testing group; Rad-Scores, radiomics scores; AD, Alzheimer’s disease; aMCI, amnestic mild cognitive impairment; HC, health control; *******, *P* < 0.001; ******, *P* < 0.01.

**CBF classifier**

The comparison of the Rad-Score in CBF classifier for identifying AD and HC, AD and aMCI, aMCI and HC were presented in Fig. S4.

The Rad-Score, which is the result of the linear combination of LR model, was calculated as below:

**AD and HC**

Rad-Score =

-0.279*L_CBF_log.sigma.2.0.mm.3D_glszm_SizeZoneNonUniformity+

-0.47*L_CBF_original_firstorder_Minimum+

-0.032*L_CBF_wavelet.HLH_glszm_GrayLevelVariance+

-0.491*R_CBF_wavelet.LHL_gldm_LowGrayLevelEmphasis+

0.45*R_CBF_wavelet.LHL_firstorder_90Percentile + -0.051

**AD and aMCI**

Rad-Score =

-0.266*R_CBF_wavelet.HHL_glcm_JointAverage+

0.484*R_CBF_log.sigma.3.0.mm.3D_glcm_Imc2 + -0.089

**aMCI and HC**

Rad-Score =

-0.027*L_CBF_wavelet.HHH_glszm_HighGrayLevelZoneEmphasis+

-0.146*L_CBF_wavelet.HLL_glszm_ZonePercentage+

-0.257*R_CBF_log.sigma.2.0.mm.3D_glcm_ClusterTendency+

-0.01*L_CBF_wavelet.HHL_glszm_LowGrayLevelZoneEmphasis+

-0.341*R_CBF_wavelet.HHL_gldm_SmallDependenceEmphasis+

0.206*R_CBF_wavelet.LLL_gldm_LargeDependenceHighGrayLevelEmphasis+

-0.078*L_CBF_log.sigma.2.0.mm.3D_glszm_LowGrayLevelZoneEmphasis + 0.031





Figure S4. Rad-Scores of the subjects in CBF classifier. **a**, Rad-Scores of AD and HC in training group; **b**, Rad-Scores of AD and HC in testing group; **c**, Rad-Scores of AD and aMCI in training group; **d**, Rad-Scores of AD and aMCI in testing group; **e**, Rad-Scores of aMCI and HC in training group; **f**, Rad-Scores of aMCI and HC in testing group; Rad-Scores, radiomics scores; AD, Alzheimer’s disease; aMCI, amnestic mild cognitive impairment; HC, health control; *******, *P* < 0.001; ******, *P* < 0.01; *****, *P* < 0.05.
