# Supplementary material for: Multiparametric hippocampal signatures for early diagnosis of Alzheimer's disease using 18F‐FDG PET/MRI Radiomics
Source: CNS Neurosci Ther. 2023 Nov 30;30(4):e14539. doi: 10.1111/cns.14539 (PMC11017421; doi:10.1111/cns.14539)
Supplement: Supplementary file 2 — Data S2. [file CNS-30-e14539-s001.docx]

**Multiparametric Hippocampal Signatures for Early Diagnosis of Alzheimer's Disease using ^18^F-FDG PET/MRI Radiomics**

The performance of the multimodal classifier was evaluated in female and male subgroups with AUC = 0.85 and ACC = 83.6% for the female subgroup and AUC = 0.94 and ACC = 90.2% for the male subgroup in the classification of aMCI and HC. The performance of the multimodal classifier was evaluated in female and male subgroups as shown in **Table S1**. The comparisons of the Rad-Score in the MRI classifier for identifying AD and HC, AD and aMCI, aMCI and HC in female and male subgroups were presented in **Fig. S5**.

Table S1. The performance of multimodal classifier in female and male subgroups

|  | Female | | | Male | | |
| --- | --- | --- | --- | --- | --- | --- |
|  | AD vs HC | AD vs aMCI | aMCI vs HC | AD vs HC | AD vs aMCI | aMCI vs HC |
| AUC | 0.99 | 0.91 | 0.85 | 0.98 | 0.87 | 0.94 |
| 95%CI | 0.98-1.00 | 0.82-0.99 | 0.76-0.95 | 0.94-1.00 | 0.76-0.98 | 0.88-1.00 |
| ACC | 95.4% | 87.9% | 83.6% | 94.9% | 85.0% | 90.2% |
| SEN | 100% | 90.6% | 76.5% | 89.5% | 78.9% | 90.5% |
| SPE | 90.9% | 85.3% | 90.9% | 100% | 90.5% | 90.0% |
| PPV | 91.4% | 85.3% | 89.7% | 100% | 88.2% | 90.5% |
| NPV | 100% | 90.6% | 78.9% | 90.9% | 82.6% | 90.0% |

AD, Alzheimer’s disease; aMCI, amnestic mild cognitive impairment; HC, health control; AUC, the area under the curve; CI, confidence interval; ACC, accuracy; SEN, sensitivity; SPE, specificity; PPV, positive predictive value; NPV, negative predictive value.


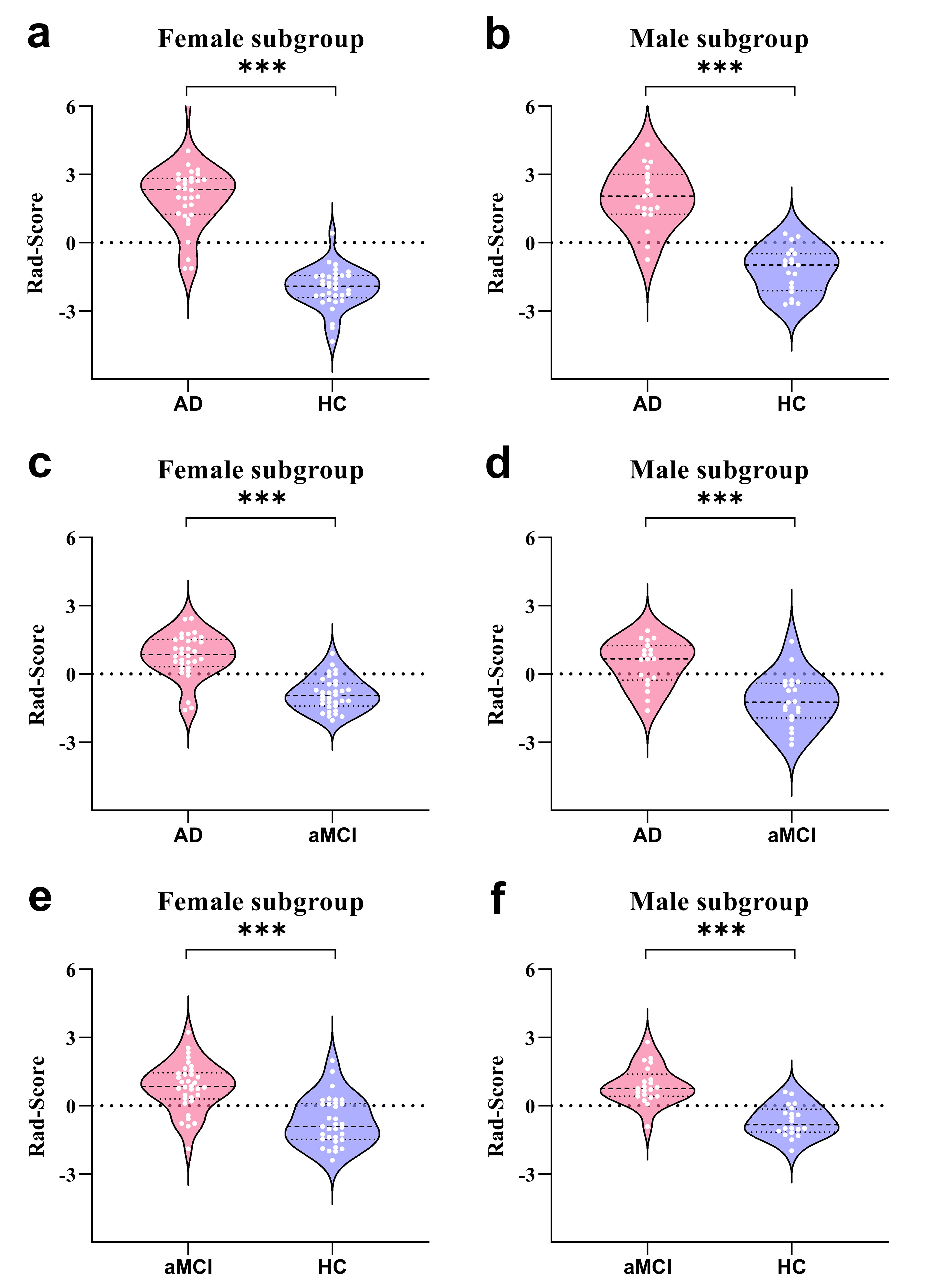


Figure S5. Rad-Scores of the subjects in female and male subgroups of the multimodal classifier. a, Rad-Scores of multimodal classifier for AD and HC in female subgroup; b, Rad-Scores of multimodal classifier for AD and HC in male subgroup; c, Rad-Scores of multimodal classifier for AD and aMCI in female subgroup; d, Rad-Scores of multimodal classifier for AD and aMCI in male subgroup; e, Rad-Scores of multimodal classifier for aMCI and HC in female subgroup; f, Rad-Scores of multimodal classifier for aMCI and HC male subgroup; Rad-Scores, radiomics scores; AD, Alzheimer’s disease; aMCI, amnestic mild cognitive impairment; HC, health control; ***, *P* < 0.001.
